# Prospective Comparison of Saliva and Nasopharyngeal Swab Sampling for Mass Screening for COVID-19

**DOI:** 10.3389/fmed.2021.621160

**Published:** 2021-02-23

**Authors:** Mathieu Nacher, Mayka Mergeay-Fabre, Denis Blanchet, Orelie Benoit, Tristan Pozl, Pauline Mesphoule, Vincent Sainte-Rose, Véronique Vialette, Bruno Toulet, Aurélie Moua, Mona Saout, Stéphane Simon, Manon Guidarelli, Muriel Galindo, Barbara Biche, William Faurous, Laurie Chaizemartin, Aniza Fahrasmane, Devi Rochemont, Nicolas Vignier, Astrid Vabret, Magalie Demar

**Affiliations:** ^1^Centre d'Investigation Clinique (CIC) Institut National de la Santé et de la Recherche Médicale (INSERM) 1424, Centre Hospitalier de Cayenne Andrée Rosemon, Cayenne, French Guiana; ^2^Département Formation Recherche (DFR) Santé, Université de Guyane, Cayenne, French Guiana; ^3^Laboratoire, Centre Hospitalier de Cayenne Andrée Rosemon, Cayenne, French Guiana; ^4^Centre de Ressources Biologiques Amazonie, Centre Hospitalier de Cayenne Andrée Rosemon, Cayenne, French Guiana; ^5^Unité Mixte de Recherche Tropical Biome and Immuno-Pathology (TBIP), Université de Guyane, Cayenne, French Guiana; ^6^Centre Délocalisé de Prévention et Soins de Maripasoula, Maripasoula, French Guiana; ^7^Service de Virologie, CHU de Caen, Caen, France

**Keywords:** COVID-19, saliva, sensitivity, PCR, nasopharyngeal

## Abstract

Current testing for COVID-19 relies on reverse-transcriptase polymerase chain reaction from a nasopharyngeal swab specimen. Saliva samples have advantages regarding ease and painlessness of collection, which does not require trained staff and may allow self-sampling. We enrolled 776 persons at various field-testing sites and collected nasopharyngeal and pooled saliva samples. One hundred sixty two had a positive COVID-19 RT-PCR, 61% were mildly symptomatic and 39% asymptomatic. The sensitivity of RT-PCR on saliva samples vs. nasopharygeal swabs varied depending on the patient groups considered or on Ct thresholds. There were 10 (6.2%) patients with a positive saliva sample and a negative nasopharyngeal swab, all of whom had Ct values <25 for three genes. For symptomatic patients for whom the interval between symptoms onset and sampling was <10 days sensitivity was 77% but when excluding persons with isolated N gene positivity (54/162), sensitivity was 90%. In asymptomatic patients, the sensitivity was only 24%. When we looked at patients with Cts <30, sensitivity was 83 or 88.9% when considering two genes. The relatively good performance for patients with low Cts suggests that Saliva testing could be a useful and acceptable tool to identify infectious persons in mass screening contexts, a strategically important task for contact tracing and isolation in the community.

## Introduction

Current testing for COVID-19 relies on reverse-transcriptase polymerase chain reaction (RT-PCR) from a nasopharyngeal swab specimen (1). Nasopharyngeal sampling requires human resources and training, personal protective equipment and swabs, and time, generating testing bottlenecks and potential exposure to transmission at crowded testing sites. Moreover, the unpleasantness of the procedure and the long waiting delays for swab collection and results may dissuade some persons to get tested or to repeat tests when they are negative. There is an urgent need for innovative testing strategies to rapidly identify cases, reduce waiting delays, and facilitate mass screening. Saliva samples have advantages regarding ease and painlessness of collection, which does not require trained staff and may allow self-sampling. The comparison of real time PCR results on salivary and nasopharyngeal samples has shown discrepancies between studies, with most finding greater sensitivity and lower RT-PCR Cts in nasopharyngeal swab samples (2–4) whereas others found greater sensitivity in saliva samples (5, 6). The sources of variation may have been the study population (hospitalized patients vs. screening of contacts or mildly symptomatic patients), saliva collection techniques and timing, conditioning and delays in processing raw saliva samples, or differences in the RT-PCR techniques used.

French Guiana is an Overseas French territory between Brazil and Suriname. Although it has a French Health System, it is isolated and its limited hospital capacity is vulnerable to the COVID 19 epidemic surge. As the epidemic peaked in July 2020, intense efforts were undertaken to expand hospital and ICU capacity, to continue contact tracing and offer a place to quarantine for patients that were unable to isolate themselves at home, and to expand COVID-19 testing and reduce testing bottlenecks at the public and private laboratories on the territory and the ensuing renouncement to get tested. We here report the first prospective study of the performance of saliva testing compared to nasopharyngeal swabs in a field context of mass screening in French Guiana.

## Methods

### Context

This French territory neighboring Amapa state in Brazil has been highly impacted by COVID-19 with 3.2% of the population having had a confirmed infection, notably among the poorest populations (7). In this context, testing and tracking were implemented throughout the epidemic, testing tents and mobile testing teams including the remote health centers, the Red Cross, Médecins du Monde, and the reinforcements from the Réserve Sanitaire were coordinated by the regional health agency to investigate around clusters of cases. The testing efforts for this small population peaked to nearly 0.5% of the population screened in a day.

### Study Conduct

Between July 22th and September 10th, we prospectively enrolled consecutive, persons aged 3 years or more with mild symptoms suggestive of COVID-19 and asymptomatic persons with a testing indication at various testing sites and mobile testing brigades in French Guiana reaching remote sites up to 240 km in the Amazonian Forest. During screening missions, mobile teams, consisting of Healthcare personnel (doctors, nurses) were coordinated by the Health Regional Agency of French Guiana, targeting villages, neighborhoods, where the virus was circulating collected persons often out of doors or in health centers. These mobile teams were made up of staff from the Red Cross, Médecins du Monde, the Cayenne hospital PASS, the Maripa Soula health center, and the health reserve. Team travel was coordinated and decided by the health regional agency of French Guiana each week during a weekly update and was guided the knowledge of current clusters of cases which triggered screening campaigns in the concerned neighborhoods—urban or rural, and usually socially disadvantaged; in addition, patients requiring hospitalization for non-COVID reasons (for example a fractured limb) were screened to rule out infectiousness; during the peak of the epidemic drive through testing services were also deployed to offer testing to any person requesting a test. Inclusion criteria were: males or females with an indication to perform a COVID diagnostic test (symptomatology, contact case, systematic screening, etc.), aged at least 3 years old. Non-inclusion criteria were refusal of the patient or his/her legal representative, person taking treatments that reduce salivary volume (anticholinergic activity), impossibility of carrying out the Nasopharyngeal swab, and persons under guardianship or curatorship, or placed under protective measures. All study participants were enrolled and sampled in accordance with the protocol. An investigator explained the objectives of the study and obtain the oral consent of the patient or his/her legal representative. The form was completed by the investigator or a person delegated by the investigator. The trained nurse present during the testing mission performed the nasopharyngeal swab and collected the salivary sputum sample in a urine container. A trained agent carried out a short questionnaire. At the end of each day, all completed forms and samples were sent to Cayenne hospital and stored at 4°C before analysis. Samples and participant information were non-individually identifiable and collected with a unique identifying number.

### Laboratory Analysis

The same technique was used for the two samples throughout the study: the QIAsymphony and GeneFinder kit, a Real-time PCR assay. GeneFinder™ COVID-19 detects SARS-CoV-2 by amplification of RdRp gene, E gene, and N gene according to WHO's recommended protocol. Viral nucleic acid was extracted by using the QIAamp DSP viral kit on the QIAsymphony RGQ, an integrated fully automated nucleic acid extraction (chemical lysis and paramagnetic bead binding) and sample preparation platform (Qiagen GmbH, Germany). The real-time PCR assays for SARS Cov2 were performed with an Applied 7500 cycler (Thermofisher) with the Genefinder kit (Ellitech group) that could detect the N gene, RdRp and E gene, which is not specific to COVID-19. As the Nucleic acid extraction methods could affect the results of viral nucleic acid amplification tests, we treated the couple saliva-nasopharyngeal specimens with the same method and most of the time in the same series, the eluates were obtained from 200 μl of specimens (300 μL – 100 μL dead volume). The remainder of each sample was divided into paired aliquots kept in a biorepository for further studies evaluating new screening tools.

### Statistical Analysis

Statistical analysis was performed using STATA® 16 (Stata corporation, College Station, Texas, USA). Cross tabulations considering different subgroups was performed. We considered the RdRp and N genes, specific for SARS-Cov2, to calculate different Ct categories. The raw data can be accessed at https://doi.org/10.7910/DVN/KPLJ9A.

### Ethical

The protocol received ethical approval from the Comité de Protection des Personnes under the number 2020-A02009-30/SI:20.07.07.54744.

## Results

We included 776 patients between July 22th and September 10th. The sex ratio (M/F) was 1.6, the mean age was 40 (standard deviation = 16.8). Overall, 61% were mildly symptomatic and 39% were asymptomatic. For symptomatic patients, 84% had a symptoms onset <10 days, and 4% were hospitalized within 2 weeks after inclusion.

### Patients With Positive RT-PCR

**Figure 1:**
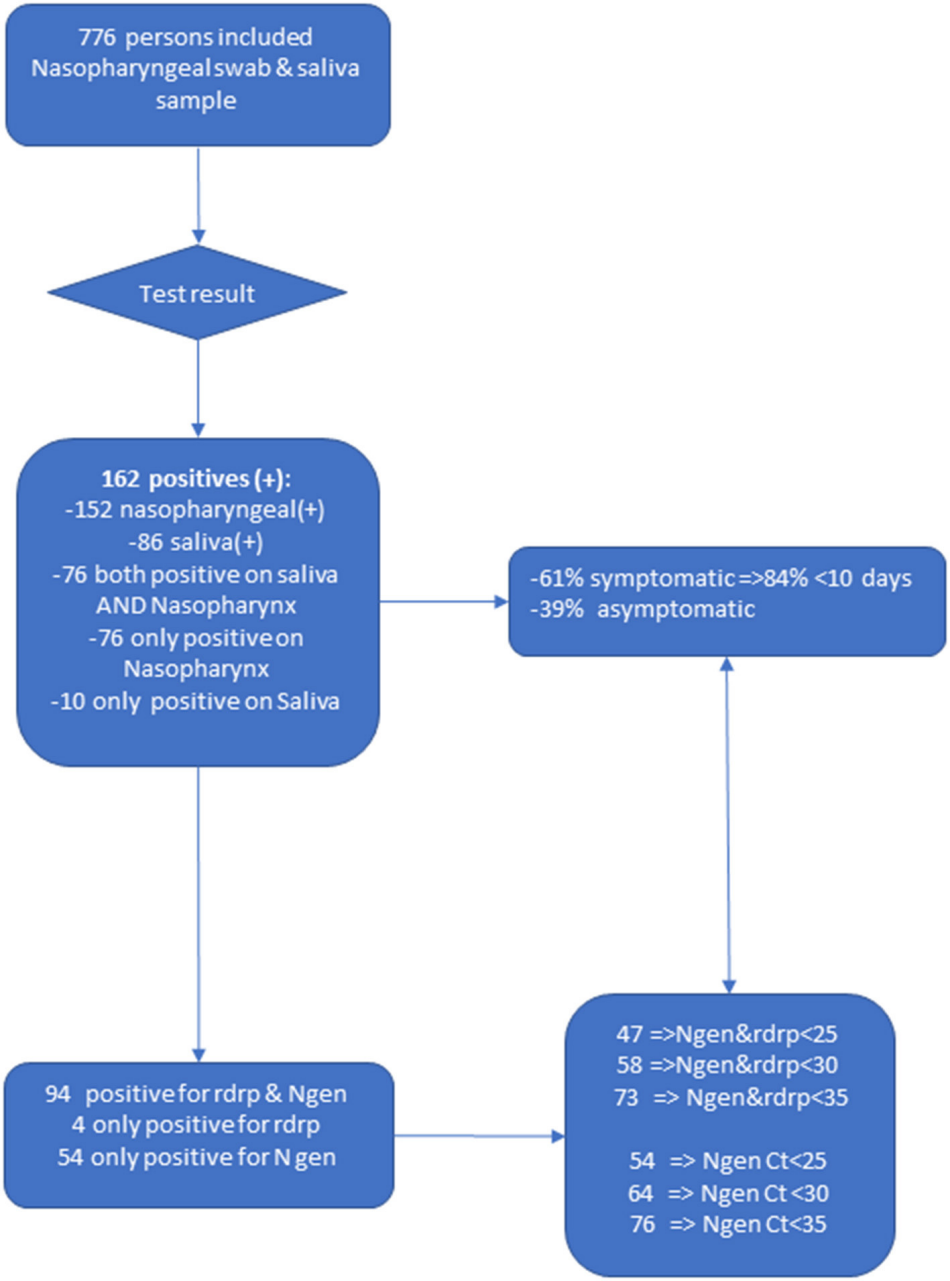
Flow chart of the Covisal study.

The crude analysis showed that 152 had a positive RT-PCR on the nasopharyngeal sample and 86 had a positive RT-PCR on the saliva sample; 76 persons had both a positive Nasopharyngeal and Saliva RT-PCR result, while 76 had a positive nasopharyngeal RT-PCR but a negative saliva RT-PCR; Finally, 10 patients (6.2%) had a negative Nasopharyngeal RT-PCR but a positive saliva RT-PCR (Figure 1). In total 162 (20.9%) of patients had a positive result on either the Nasopharyngeal or the saliva sample.

### Sensitivity, Symptoms, and Ct Values

**Figure 2:**
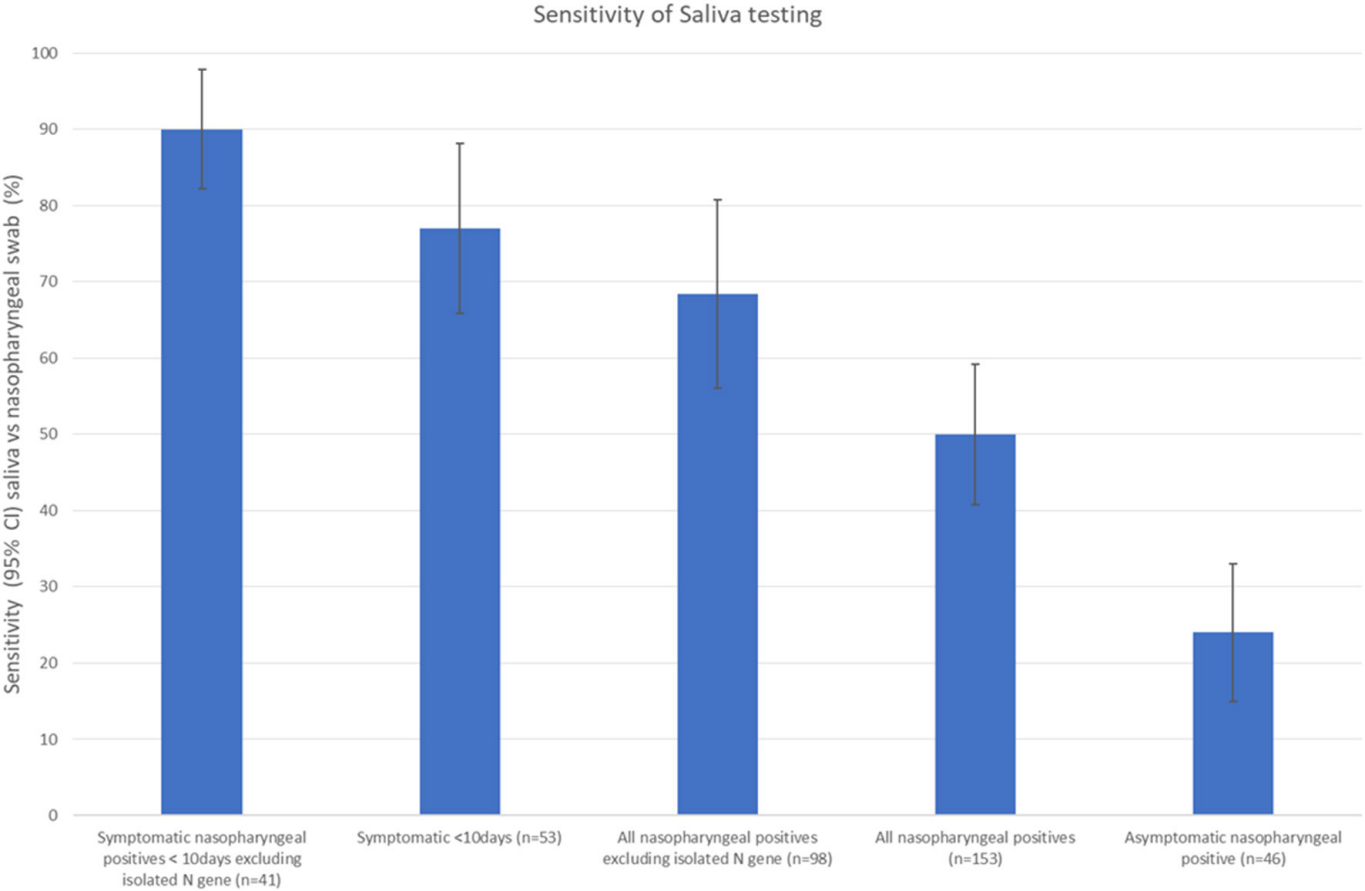
Sensitivity of saliva testing vs. nasopharyngeal swabs for RT-PCR for different groups in a community screening context.

**Figure 3:**
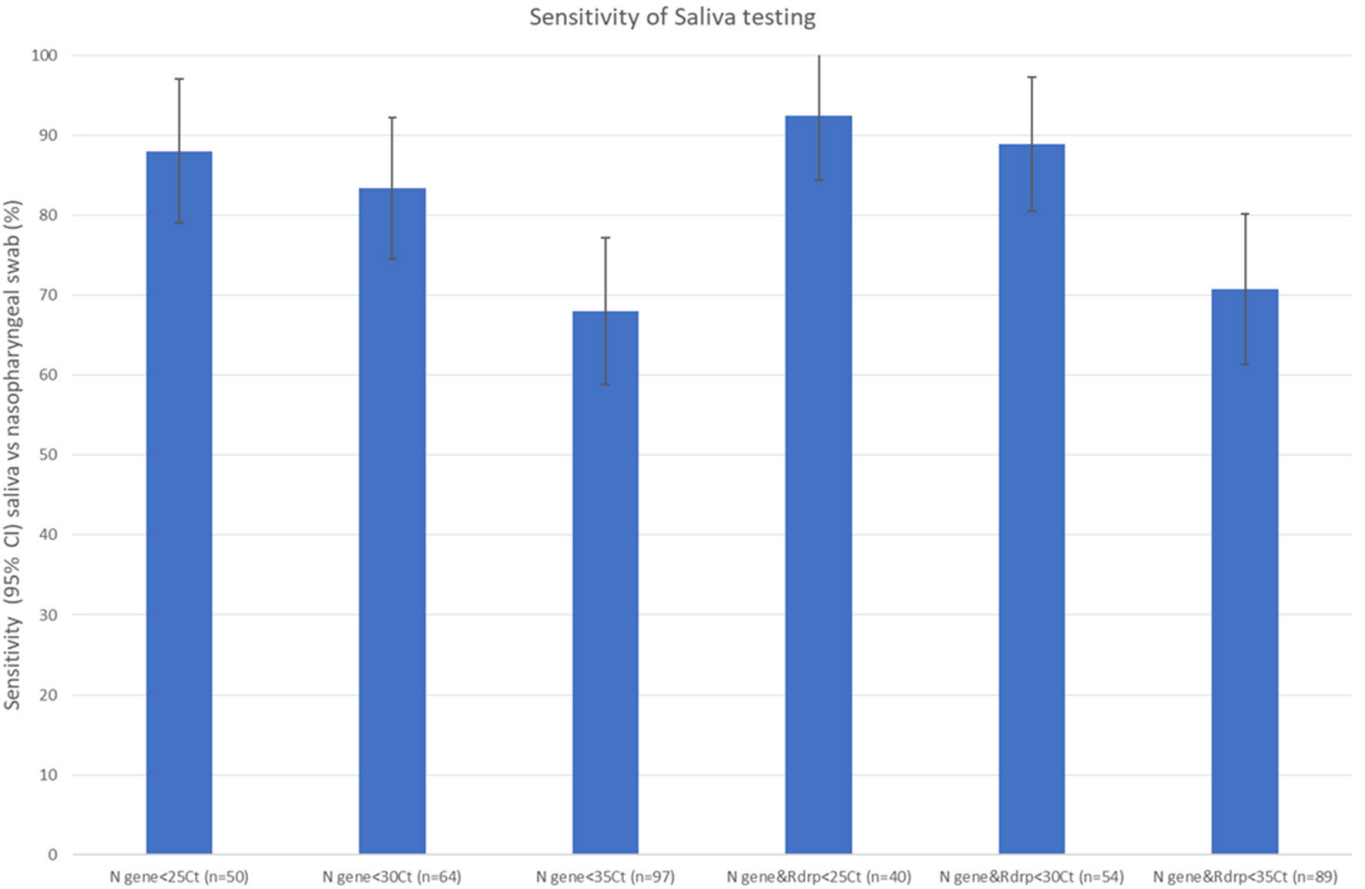
Sensitivity of saliva testing vs. nasopharyngeal swabs for different RT-PCR Cts in a community screening context.

The sensitivity of RT-PCR on saliva samples vs. nasopharygeal swabs varied depending on the patient groups considered (Figure 2) or on Ct thresholds (Figure 3). When considering all patients with at least one gene amplification—irrespective of delays, symptoms, or Cts, sensitivity was low (50%); For symptomatic patients with an interval between symptoms onset and sampling under 10 days sensitivity was 77%; however, when excluding persons with isolated N gene positivity (54/162) from this subgroup, sensitivity was 90%.For asymptomatic patients, the sensitivity was only 24%, the lowest of all studied groups (Figure 2).

**Figure 4:**
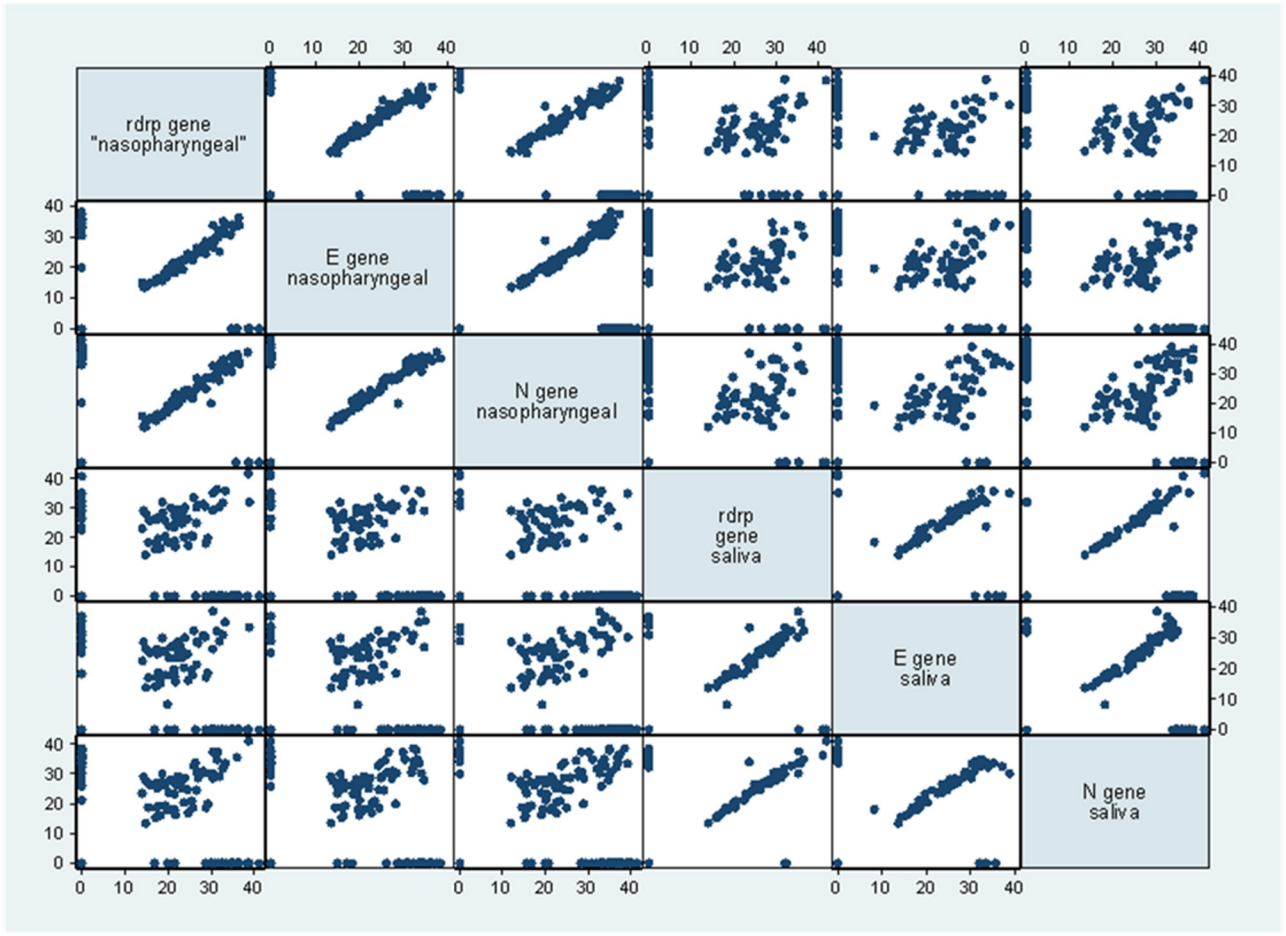
Scatterplot matrix for the Ct of different genes in the nasopharyngeal and saliva samples. There was a “fanning” pattern with greater dispersion at higher Ct values for different genes in the nasopharyngeal and saliva samples.

Recent studies have argued that transmission potential -estimated by the capacity to infect cell cultures- was restrained to those with low Cts (8, 9), a proxy for high viral load. When we looked at patients with Cts <30, sensitivity was 83 or 88.9% when considering two genes. Among the 10 patients with a positive saliva sample and a negative nasopharyngeal swab, all had Ct values <25. Figure 4 shows increasing dispersion for the higher Ct values of the nasopharyngeal vs. saliva sample scatterplots for the different genes amplified by RT PCR emphasizing the greater discordance between samples among patients with lower viral loads.

## Discussion

Contrarily to two studies suggesting a greater positivity rate for saliva (5, 6), we observed that saliva testing was less sensitive than nasopharyngeal swabs. Whereas, most studies were hospital-based collecting saliva in the early morning before mouth rinsing and breakfast, our study was a screening study that was performed in difficult field conditions targeting hard to reach populations after breakfast and teeth brushing, moreover out of doors in a tropical context. These realistic conditions were however also a limitation because of the heterogeneity of inclusion sites. Since the main objective was to compute sensitivity, in order to shorten the time allocated to each inclusion, there was limited clinical/epidemiology data from tested individuals and no data on possible repeated testing. The study started after the epidemic peak and hence inclusion of positive patients became increasingly difficult, and the number of positives was hence insufficient to conduct stratified analyses on subgroups, and particularly for asymptomatic persons with positive RT-PCR who may pool active infections and residual shedding but no clear time frame that could allow to disentangle the two. The poor sensitivity on asymptomatic positive nasopharyngeal swabs was thus presumably also linked to the inclusion of non-infectious patients in the denominator. A third of positives only had a positive N gene, the RdRp and E gene being negative. Based on the empirical experience of the laboratory, such patients were considered to be at later stages of the infection.

The relatively good performance for patients with low Cts suggests that Saliva testing could be a useful and acceptable tool to identify infectious persons in mass screening contexts, a strategically important task for contact tracing and isolation in the community. With the considerable testing bottlenecks, alleviating the workload and shortening the sample collection time would be improvements that could reduce waiting times to get tested and human-resource costs. The sensitivity saliva samples for asymptomatic persons seemed insufficient but without any temporal indication about the onset of infection, it should be further studied by Ct values with a larger sample size. In view of the present results the French Health authorities have officially declared that saliva testing may be used on symptomatic patients only when nasopharyngeal tests cannot be used (10).

## Data Availability Statement

The raw data supporting the conclusions of this article will be made available by the authors, without undue reservation.

## Ethics Statement

The studies involving human participants were reviewed and approved by Comité de Protection des Personnes. Written informed consent to participate in this study was provided by the participants' legal guardian/next of kin.

## Author Contributions

MN and MD: conception. MM-F, DB, OB, TP, PM, VS-R, VV, BT, AM, MS, SS, MGu, MGa, BB, WF, LC, AF, DR, NV, AV, and MD: investigation. MN: analysis and first draft writing. MN, MM-F, and MD: review and editing. All authors contributed to the article and approved the submitted version.

## Conflict of Interest

The authors declare that the research was conducted in the absence of any commercial or financial relationships that could be construed as a potential conflict of interest.
